# Interventions for promoting physical activity among European teenagers: a systematic review

**DOI:** 10.1186/1479-5868-6-82

**Published:** 2009-12-06

**Authors:** Femke De Meester, Frank J van Lenthe, Heleen Spittaels, Nanna Lien, Ilse De Bourdeaudhuij

**Affiliations:** 1 Department of Movement and Sport Sciences, Faculty of Medicine and Health Sciences, Ghent University, Ghent, Belgium; 2 Department of Public Health, Erasmus University Medical Center Rotterdam, Rotterdam, the Netherlands; 3 Department of Nutrition, Faculty of Medicine, University of Oslo, Oslo, Norway

## Abstract

**Background:**

Although physical activity is considered to yield substantial health benefits, the level of physical activity among European teenagers is not sufficient. Adolescence is characterized by a decline in physical activity level. Many studies investigated the effectiveness of interventions promoting physical activity among young people, but none dealt with the available evidence specific for Europe. This review was conducted to summarize the effectiveness of interventions to promote physical activity among European teenagers.

**Methods:**

A systematic review was conducted to identify European intervention studies published in the scientific literature since 1995. Four databases were searched, reference lists were scanned and the publication lists of the authors of the retrieved articles were checked. The ANGELO framework was used to categorise the included studies by setting and by intervention components.

**Results:**

The literature search identified 20 relevant studies. Fifteen interventions were delivered through the school setting, of which three included a family component and another three a family and community component. One intervention was conducted within a community setting, three were delivered in primary care and one was delivered through the internet. Ten interventions included only an individual component, whereas the other ten used a multi-component approach. None of the interventions included only an environmental component.

Main findings of the review were: (1) school-based interventions generally lead to short term improvements in physical activity levels; (2) improvements in physical activity levels by school-based interventions were limited to school related physical activity with no conclusive transfer to leisure time physical activity; (3) including parents appeared to enhance school-based interventions; (4) the support of peers and the influence of direct environmental changes increased the physical activity level of secondary school children; (5) the assumption that a multi-component approach should produce synergistic results can not be confirmed; (6) when interventions aimed to affect more than one health behaviour the intervention appeared to be less effective in favour of physical activity.

**Conclusion:**

Overall, the current European literature supports the short-term effectiveness of school-based physical activity promotion programmes. The available evidence for the effectiveness in other settings is rather limited and underscores the need for further research.

## Introduction

Physical activity has been put forward as an important and modifiable factor influencing people's health. Participation in regular physical activity has been associated with substantial health benefits [1]. Moreover, physical activity has an inverse linear relationship with obesity and many chronic diseases namely cardiovascular diseases, different types of cancer, diabetes mellitus type 2 and osteoporosis [2-4].

In adolescence, health behaviours are developed and these behaviours may persist into adulthood [5,6]. Unfortunately, this period of life is marked by a significant decline in the time spent in physical activity [1,7,8]. Despite the numerous health gains related to regular physical activity, most of the teenagers in Europe do not achieve the public health guidelines [9-11]. These guidelines state that 60 minutes or more of at least moderate physical activity per day is enough to provide substantial health benefits [12,13]. The development of effective interventions for the promotion of physical activity among teenagers is therefore a priority in current public health research. Recent systematic reviews summarized the evidence of the effectiveness of interventions promoting physical activity among young people [14-19]. However, these previous reviews did not address the effects on teenagers separately [14-16] and they did not incorporate an assessment of the methodological quality of the studies [14-18]. In addition, none dealt with the available evidence specific in Europe [14-19]. As Europe is different from other continents concerning the environmental, cultural, social, economical and political conditions, it is still unclear what intervention methods are successful among European teenagers. Regarding health promotion, specific differences between Europe and other continents include the priority of health concerns, the structure and organization of education, environmental features at home and at the neighbourhood level and the beliefs and attitudes concerning physical activity and its relation with health. As a consequence, the public health guidelines on physical activity interventions specific for Europe must be in line with the characteristics and needs of this continent.

The purpose of this review is to give an overview of the current interventions that target physical activity in European teenagers. For this purpose, we will use a conceptual framework in order to give a structured overview and to allow some comparison between the results of the interventions. The ANalysis Grid for Environments Linked to Obesity (ANGELO) framework was chosen to categorise the studies by setting (school setting, school setting with involvement of family, school setting with involvement of family and community, community with involvement of schools, primary care and individual setting) and to classify the intervention components (individual, physical, socio-cultural, economical and political components). This framework was specifically conducted to conceptualize obesogenic environments and to identify potential intervention settings and strategies [20].

With the information given in this systematic review we aim to supply policy makers and health care providers with an evidence based summary in order to (1) build a bridge across theory and practice, (2) offer new ideas and recommendation for a variety of settings and sectors and (3) guide future research in this area.

## Methods

### 1. Search strategy

For the purpose of this review a literature search was conducted to identify interventions to promote physical activity in teenagers. A structured electronic bibliographic database search was used to retrieve articles published in the English language between January 1995 and May 2008. Four databases were searched (e.g. Medline (Pub Med), Web of Science, SPORT Discus, Cochrane library) using a search filter comprised of a group of elements describing: outcome behaviour (e.g. sports, exercise, physical activity), population (e.g. teenager, adolescent, child), intervention (e.g. primary prevention, health education, promotion), and study design (e.g. controlled trial, evaluation studies). The search filter included subject headings (MeSH) and a range of free text words. To ensure the search strategy would capture all relevant studies, enough time had been taken to test the search filter and to adapt it in an appropriate, designated way for each database.

### 2. Selection process

The search strategy resulted in a list of potentially relevant studies. In order to be included, the articles were screened to determine if they met the postulated inclusion criteria. The review was restricted to articles on studies conducted in Europe since 1990 and published in scientific literature since 1995. The latter in order to particularly focus on interventions conducted in contemporary epidemiological and environmental circumstances. Studies were included if (1) the effect of primary interventions to promote physical activity was evaluated, (2) a comparison or control group was used, 3) the participants were teenagers with an average age between 10 and 19 years, (4) the main outcome or one of the outcomes was an objective or self-reported measure of physical activity (e.g. actual behaviour change) and (5) an effect of the outcome measure was available on at least one follow-up measurement. In addition, multi-component interventions with other modalities (e.g. diet, smoking, alcohol) were also included. Studies were excluded if (1) the participants were individuals with a health problem or specific disease and (2) the only outcome was physical activity in education classes or physical fitness. These exclusion criteria were used to narrow the literature search to primary prevention studies promoting healthful behavioural habits and an active lifestyle. To address fully the area and to make a contribution to the state of the scientific literature, we did not limit this review to randomized controlled trials and included a wide range of intervention studies.

In a first step of the selection process the list of titles was scanned and irrelevant articles were excluded. In a next step, the abstracts were read and the full text of the remaining studies were retrieved. Subsequently, all papers were read and screened to come to a final selection. In order to maximize the yield of the search and to identify studies that have been missed in the database search, the reference lists of the retrieved articles, literature reviews and other relevant publications were scanned. At last, the publication lists of the authors and co-authors of the retrieved articles were checked. Any doubts in the inclusion/exclusion process were resolved through discussion with a second researcher.

### 3. Methodological Quality

All studies meeting the inclusion criteria underwent quality assessment using a standardized framework (Quality assessment Tool for quantitative studies, EPHPP 2008) [21] recommended by the Cochrane reviewers handbook [22]. The quality of the studies was rated on eight methodological dimensions: selection bias, study design, confounders, blinding (e.g. blinding of the participants and intervention providers), data collection methods and withdrawals and dropouts. Based on the content of the manuscripts each component was assessed and rated according to a three-grade scale: strong, moderate or weak. To obtain a global rating, the ratings were summed according to the guidelines of the quality assessment tool [21]. Two researchers independently assessed the quality of the included studies. Disagreements between the two reviewers were discussed until consensus was reached. The opinion of a third researcher was consulted to come to agreement in case of indecisions.

### 4. Data extraction and data synthesis

Data of the final sample of the studies meeting the inclusion criteria was extracted and summarized in additional file 1 and additional file 2. For the purpose of this review the included studies were tabulated according to the ANGELO Framework. This framework was specifically developed to conceptualize obesogenic environments and to identify potential intervention settings and strategies [20]. The basic framework is a 2 × 4 grid which comprises two sizes of environment (micro and macro) on one axis and four types of environment on the other. First, the included studies were categorized by intervention setting (school setting, school setting with involvement of family, school setting with involvement of family and community, community with involvement of schools, primary care and individual setting). Further, the environmental intervention components were classified as physical (what is available), socio-cultural (what are the attitudes and beliefs), economical (what are the costs) or political (what are the rules) [20]. The framework was extended with an overview of the individual intervention components. In addition, the training provided for the main providers and/or the parents is also described in additional file 1.

The use of the ANGELO framework in this review will ensure that all intervention components will be considered and that the evidence in this field will be conceptualized in a consistent way. A brief description of the methodological details of the interventions, such as study design, target population, relevant outcomes related to behaviour change, duration of the intervention, the main results and the ratings of the quality assessment process are given in additional file 2. In both additional file 1 and additional file 2 the studies were categorised in accordance to their ratings of the quality assessment process.

In the results section the outcomes of the included studies are summarised by type of intervention setting and related with the intervention components identified by the ANGELO Framework. Because of the heterogeneity of the studies with respect to the outcome measures for physical activity, study design and study sample, a meta-analysis was not performed.

## Results

### 1. Results search strategy

The combined search strategy produced approximately 6900 potentially relevant articles. Based on the titles, irrelevant publications and duplicates were eliminated, resulting in 351 studies. After reading these abstracts, 68 studies were selected as still potentially relevant and retrieved in full text. A careful review of these manuscripts resulted in 14 interventions meeting the above mentioned inclusion criteria. Reference checking of these 14 yielded another two studies, in addition to four studies found in the reference lists of relevant reviews. Thus, the final review includes a total of 20 interventions.

The majority of excluded articles were studies conducted in the US and studies not evaluating a physical activity intervention. Other important reasons for exclusion were the lack of a physical activity measure or targeting a different age group.

### 2. Methodological quality included studies

After initial assessment of the methodological quality, the two reviewers reached an inter-rater agreement from 55% to 89% on the six items. An incomplete description of the study and reading or interpretation errors were the cause of the majority of the disagreements. Consensus was reached on all items except for 'blinding'. The interpretation of this item led to a strongly divergent rating (55% proportion of agreement) and a third reviewer was consulted to reach a consensus on this item.

The results of the quality assessment are summarised in additional file 2. The global rating of two studies [23,24] was strong, eight studies [25-32] were rated as moderate and ten studies [33-42] were rated as weak.

Overall the study designs were strong. Sixteen interventions [23-32,34,35,38,40-42] were controlled trials with a no-intervention control group. Of these studies, 11 [23,26-29,31,32,38,40-42] reported randomization to allocate the units to condition but only three [28,41,42] described the randomization procedure. The majority of the studies used clusters of units such as schools [24,26,27,29-31,34,35,39,40], classes [23,28] and communities [25], rather than individuals [32,33,37,38,41,42] to allocate to the conditions.

In six studies [23,28,32,33,37,38] contamination between the control and intervention group was possible. In almost half of the studies [23,24,26,27,29,32,35,38] there were no significant differences between the conditions at baseline or possible confounders were controlled for by stratification or matching. Furthermore, in all the studies except one [32], the intervention providers were aware of the exposure status of the participants and in only two of these studies [28,31] the participants were informed about the purpose of the intervention after the post-test.

A variety of outcome measures of physical activity was reported. The most common assessment methods were self-report questionnaires or recall instruments. Only one study [31] used an objective measure of physical activity, while the results of another study [27] relied on the combination of self report and objective measures in a subsample. Ten studies [23,24,26,27,29-33,38] used valid and reliable instruments to measure physical activity. In the other studies [25,28,34-37,39-42] validation and/or reliability was either not tested or not described.

Drop-out percentages were mentioned in or could be calculated for fifteen studies [23-25,27-29,31,32,36-42]. Drop-out ranged from 2,6% [38] to 66% [42]. The most common reasons of dropouts were non response, absence on the day of the measurement and questionnaires filled in inaccurately.

### 3. Study findings

The effectiveness of the interventions regarding physical activity is reported in additional file 2, in the following section the results are summarised using the ANGELO framework.

### School

Of the 20 included studies, nine studies [23,28,30,31,33,34,37,38,40] were conducted in school environments without involvement of the family or community. Only two [31,37] targeted primary school children. The first one, the study of Verstraete et al. [31], a randomized controlled trial, investigated the effect of providing game equipment. Due to an incomplete description of the blinding process, this study was given a moderate quality rating. At the end of the 3-month intervention, the accelerometer data clearly indicated that during lunch break the intervention was effective in increasing physical activity in boys and girls. During morning recess the intervention increased only girls' moderate physical activity. This intervention was part of a broader physical activity promotion programme what may have implications for the interpretation of the results.

In contrast, the second study, the 3 month health educational program 'An adventure with Pelle Pump' evaluated by Lindberg et al. [37] did not affect the actual health behaviour of 10-year olds. This Swedish study targeted physical activity, diet and smoking. The design of the study and the lack of information on the validity and reliability of the questionnaire accounts for the low quality rating of this study.

The remaining seven studies [23,28,30,33,34,38,40] were carried out in secondary or high schools, one was given a high quality rating [23], two were given a moderate quality rating [28,30] and four were given a low quality rating [33,34,38,40]. The moderate and low quality ratings reflect an incomplete description of the dropouts [30,33,34] and of the validity and reliability of the assessment tool [28,34,40], a less rigorous design [38], lack of blinding of the participants and intervention providers [33,34,38] and an inappropriate manner to control for confounders [40]. Five studies [23,28,30,33,38] were restricted to the implementation of individual intervention components.

In the first study, Tsorbatzoudis et al. [30] evaluated a 12-week health educational program implemented in physical education lessons in a Greek junior high school resulting in positive changes in exercise habits (F = 14.04, p < 0.001). These findings could not be sustained 4-6 weeks after the end of the intervention.

A second study from the United Kingdom, conducted by Lubans and Sylva [38], evaluated a health educational program combined with an exercise program. At the end of the 10-week intervention, the time spent in moderate to vigorous physical activity increased significantly in the intervention group while it decreased in the control group (F = 9.69, p = 0.001). Three months after the intervention, a significant difference between the intervention and control group was not longer present.

Third, Hill et al. [28] evaluated the effectiveness of a theory-based persuasive leaflet augmented with two cognitive change techniques resulting in 3 intervention conditions. The participants were students attending a secondary school in South-East England. Three weeks after the intervention, the reported exercise of the teenagers in the three leaflet conditions increased significantly compared to the control condition. The leaflet conditions did not differ significantly in their impact.

A fourth study examined the effectiveness of a computer-tailored intervention during classes. Three months after the intervention, this 1-hour high quality, Belgian study of Haerens et al. [23] showed a positive impact on school related physical activity. No effects were found for leisure time physical activity nor for total physical activity.

The fifth study was conducted in the United Kingdom by Chatzisarantis and Hagger [33] and compared a persuasive message targeting modal salient beliefs with a persuasive message targeting nonsalient behavioural beliefs. No positive results were found in this brief study.

The two remaining secondary school-based studies of Murphy et al. [40] and Digelidis et al. [34] implemented some environmental changes in addition to an individual intervention component. Both trials were rated as weak, only the Irish study of Murphy et al. [40] was effective. This study investigated a teacher-led physical activity program versus a traditional self-led osteogenic physical activity program in inactive teenage girls. At the end of the 6-month intervention both groups were moved from the least to the most active quartile of the age-matched population. The girls in the no intervention control group did not change. One month after the intervention the process evaluation of a subsample revealed that the girls who followed the self-led program were still physically active in contrast to the girls who followed the teacher-delivered program.

The last school based study, the Greek study of Digelidis et al. [34] targeted physical activity and nutrition simultaneously. The combination of (1) a health educational program and (2) changes in the socio-cultural and political school environment did not succeed in positive changes in the leisure time physical activity behaviour.

### School with involvement of family

Three intervention studies evaluated an individual approach with environmental changes on school and family level. The study from Harrison et al. [24] and the study from Christodoulos et al. [26] targeted teenagers at primary school level, while the study from Haerens et al. [27] was implemented in a secondary school. All three studies showed positive changes in the level of physical activity. Based on the content of the published manuscripts, the studies were given an acceptable quality rating. The study from Harrison et al. [24] was rated as strong and the studies from Christodoulos et al. [26] and Haerens et al. [27] were rated as moderate.

The first study, the 16-week Irish 'Switch off-get active' program of Harrison et al. [24], succeeded to increase the values for moderate to vigorous activity in lower socioeconomic groups. The program combined an individual educational approach including simple behaviour modification techniques with parental support.

In the second study of Christodoulos et al. [26], the Greek primary school children reported at the end of the intervention more time spent in out of school organised physical activities (p < 0.020) and in moderate to vigorous physical activity (p < 0.064) than pupils from the control school. In addition, more pupils reached the recommendations of 60 minutes of moderate to vigorous physical activity daily (77.4% v 55.1%, p < 0.043). The small number of participants in a single school district must be taken into account to consider the results and the transferability of the results.

In the third study, middle school teenagers were targeted and the intervention [27] showed differences in effects according to the context of the activities. A personal tailored intervention combined with changes in the physical, socio-cultural and political environment induced a larger impact on school related physical activity (p < 0.05 in boys) than on leisure time physical activity (n.s.). Furthermore, the accelerometer data of a subsample demonstrated that the Belgian intervention prevented a decrease of the level of moderate to vigorous physical activity in boys (p < 0.08) while in girls it increased in both the intervention and control group (n.s.). The effects found after the first intervention year were almost similar with those after the second year and parental involvement did not result in additional effects [43].

### School with involvement of family and community

One elementary and two secondary school trials involved the family and the wider community. The first study, the programme 'JUMP-in, kids in motion' of Jurg et al. [35] was implemented at elementary school level and involved children from low socio-economic backgrounds. This Dutch programme incorporated an educational component combined with changes in the physical, sociocultural and political environment. It succeeded in: (1) preventing grade 6 children from becoming less active (2) grade 6 children were more likely to meet the postulated guidelines. No significant changes were found in grade 4 and grade 5. This study failed to blind the participants and intervention providers. Furthermore, the questionnaire used was not tested on validity and reliability and no detailed information was available on withdrawals and dropouts. Given these limitations, the study was rated as weak.

The two other interventions known as the 'Intervention centred on adolescents' physical activity and sedentary behaviour (ICAPS)' and the 'Wessex Healthy schools Award' were evaluated in secondary school children by Simon et al. [29,44] and Moon et al. [39]. Both programmes combined a health educational program and changes in four environmental components. The intervention of Simon et al. targeted physical activity while the study of Moon et al. targeted physical activity, diet and smoking. Although both studies consisted of the same intervention components, the content of these components, the methodological quality rating of the trials and the impact on the level of physical activity of the teenagers differed a lot. The target population of the multilevel ICAPS intervention were students of middle schools in Eastern France. After the first six months the proportion of teenagers engaged in out-of-school organised physical activity increased from 64% to 83% whereas it was unchanged among the control students (p < 0.01). Since ICAPS was designed to take place over 4 academic years, the evaluation of the long-term effectiveness should provide us with more information in the future. In contrast, the study from Moon et al. [39], the 'Wessex Healthy Schools Award', that targeted three health behaviours, produced no significant changes in the physical activity level of teenagers. The study by Simon et al. [29] was rated as moderate, while the study of Moon et al. [39] was rated as weak. Although both studies were given a low rating on blinding in the quality assessment, this was the only item for the study of Simon et al. that received a low rating. The study of Moon et al. failed also on the items "confounders" and "data collection methods" due to incomplete description of these items in the published manuscripts.

### Community with involvement of school

The project 'Action Heart' developed by Baxter et al. [25] evaluated an overall community project in the United Kingdom. The project involved schools and aimed to modify three risk factors: smoking, diet and exercise. Beside the implementation of individual intervention components, the project strived for changes in the physical, socio-cultural, economical and political environment. After 3-years of health promotion work only four percent more teenagers exercised three or more times a week. The quality of this study was rated as moderate.

### Primary care

One British [42], one Irish [36] and one Spanish study [41] was carried out in a primary care setting. Particularly due to the lack of information on intervention details in the published manuscripts all three studies were rated as weak. The short counselling session in both, the Galway Health Project of Kelleher et al. [36] and the study of Walker et al. [42] did not produce apparent results for the physical activity outcomes. However, the study of Ortega-Sanchez [41], showed significant positive results. This intervention involved three counselling sessions according to the teenagers' current levels of physical activity over a 12-month period. After 6 months, the prevalence of physical activity behaviour (p = 0,008), as well as duration (p = 0.016), frequency (p = 0.010) and intensity (p = 0.007) of exercise and/or sport were higher in the intervention group compared with the control group. This effect was sustained and even more pronounced after 12 months. The level of physical activity of the teenagers was the sole focus of this intervention, while in the study of Kelleher et al. and Walker et al. the physical activity intervention was part of a multiple risk factor intervention. In additon, both studies reported a remarkable high percentage of individuals who dropped out before the end of the intervention.

### Individual mail-delivered intervention

Only one of the 20 included studies evaluated a personal mail-delivered educational program. This randomised controlled trial, conducted by Woods et al. [32] in Scotland, was made up of two packages on active living with self-instructional messages to motivate sedentary students to become more physically active. After 2 mail-delivered messages, the students of the intervention group participated significantly more in sport activities in comparison to the control group (62%; 38%, p < 0.001). The study was limited by its response rate for which it received a moderate quality rating. Only half of the baseline subjects completed the follow up questionnaire.

## Discussion

With this systematic review we aimed to identify interventions to promote physical activity among European teenagers. This review systematically identified 20 studies. The results of 13 [23,24,26-32,35,38,40,41] interventions showed positive effects on the level of physical activity of teenagers. Eleven of those studies [23,24,26-31,35,38,40] were carried out in a school setting.

A first major finding of this review was that the majority of the school-based interventions did find positive improvements in the level of physical activity [23,28,30,31,38,40]. But overall, these positive improvements were short term benefits. In the studies that included a follow-up measurement, the obtained positive intervention effects could not be sustained [30,34,38]. The small number of studies that included a follow-up measurement highlights the need for future research to focus on the long term effectiveness of school-based interventions. A second major finding was that the positive improvements generally were limited to school related physical activity and that there was no conclusive transfer to leisure time physical activity [23,33,34]. The lack of behaviour change during leisure time may have been caused by the mechanisms used in the school-based programs. Although most school-based programs intended to achieve a change in lifestyle habits and not intended to restrict their intervention to changes in school related physical activity, interventions in a school setting can unintentionally have focused more on the mechanisms of behaviour change for school related physical activity. Another highly plausible explanation is the lack of involvement and support of family and peers in the interventions. This assumption is supported by the favourable results of the school-based interventions that did involve the family on the level of physical activity during leisure time [24,26,27]. After all, parents are important gatekeepers of children's physical activity level during leisure time. Preceding research [45] has identified parental support and encouragement as correlates of the physical activity level of teenagers. In addition, parental provision of transportation to activity locations also showed a clear association with out-of-school physical activity in South Californian teenagers [46]. This confirms the third major finding: physical activity promotion strategies with parental involvement might have a greater likelihood of success. Further European research is needed to strengthen and confirm this finding.

A fourth major finding was that intervention strategies that also involved the wider community in the school-based programs, showed encouraging results [29,35,39]. The physical activity level of secondary school children increased under the influence of peers and the involvement of the community. This finding is based on the results of only two interventions [29,35]. The importance of peers and links with the wider community in the promotion of physical activity must be confirmed by urther research.

Further, there is little evidence of the effectiveness of whole community interventions with involvement of schools. Nevertheless, taking into account that community-based interventions can reach a whole population and may have a substantial impact, this approach deserves further exploration.

From the evidence found in three studies describing brief primary care based interventions we can conclude that counselling sessions delivered by a physician are promising and can have a positive effect on teenagers' physical activity when physical activity is the sole focus of the intervention [36,41,42]. We must keep in mind that the quality of those three studies was rated as weak. But, this low quality rating was particularly due to the lack of information on intervention details in the published manuscripts. To confirm this conclusion further research with high quality studies is necessary.

The 20 interventions included, varied not only in setting but also in their content, their components and as a consequence in their results. Half of the interventions [23,28,30,32,33,36-38,41,42] were single component interventions, only targeting changes on the individual level. The other interventions were multi-component interventions, including a combination of an individual behaviour change approach and some broader environmental changes (physical, socio-cultural, economical or political changes). Although we hypothesised that this multi-component approach should produce synergistic results, a fifth major finding is that we found no conclusive affirmation for this assumption.

Most of the multi-component programmes combined several environmental components with an individual intervention component. We did not find any intervention evaluating only environmental changes to promote physical activity. This makes it difficult to disentangle the most effective intervention components, or in other words, the answer to the question which components are necessary and effective and to what extent is not available yet.

Although the essential components for success should be confirmed by future research, the following approaches might be promising. Positive effects on physical activity levels in primary school children, or 10-11 year olds, were reached by providing game equipment during recess and lunch break [31] and by programs combining health education lessons and changes in environmental components such as: parental involvement and support through homework assignments or through a supervising function and inclusion of more cooperative, enjoyable, fitness and goal oriented activities in the physical education lessons [24,26,31,35,37]. Individual intervention components delivered in secondary schools that succeeded in altering teenagers' level of physical activity were: personal physical activity advice, health education to improve the students' knowledge, a counselling session adapted to the current activity level of the teenagers or an activity program. Furthermore, examples of physical, socio-cultural, economical and political intervention components that might encourage to be more physically active are: the provision of more PA opportunities (e.g. more material, more activities, more variation), stimulation of active transportation, support by parents and peers, free of charge entry to safe accessible facilities, change in public transport, partnerships with multiple parties [23,27-30,32,38,40,41].

A factor that also appeared to have an influence on the results was the target behaviour of the intervention. Less positive results were found in interventions that targeted also other health behaviours next to physical activity [25,34,36,37,39,42]. This comprises our sixth major finding: when interventions aimed to affect more than one health behaviour the intervention appeared to be less effective in changing physical activity.

The majority of the studies reviewed, are hampered by some methodological flaws. An important factor that must be considered is that the quality assessment was solely based on the content of the published manuscripts. Since not all manuscripts have described the intervention content and implementation in detail and space restrictions imposed by the journals may result in the omission of most study details, this may have led to an underestimation of the quality of the studies. One of the characteristics assessed by the quality assessment tool used in this study is 'blinding'. Although, blinding in behavioural research is certainly not always convenient or possible, it is necessary to reduce bias. Nevertheless, it should be possible to attain blinding at different study levels across intervention providers and participants. To ensure blinding it could be advisable to plan the study precisely before the onset, to inform key persons and participants about these matters and to consider back up plans for situations where blinding is threatened [47]. Unfortunately blinding was often not described in detail in the manuscript.

The variety of outcome measures used, relied mostly on self-reports. Moreover, the validity and reliability of several of the used instruments were either not described or not tested and almost all instruments measured different or slightly different constructs. Although self-report instruments can be useful in interventions with a large number of participants and multiple points of assessment, it is advisable to use more objective and standardised instruments to obtain more verifiable and comparable results.

In some school-based studies contamination may have occurred as a result of the control and intervention classes or individuals that were located in the same schools [23,28,32,33,37,38]. This could have led to an underestimation of the effectiveness of the interventions or classification of an effective intervention as ineffective because the observed results were not statistically significant.

Furthermore, the post-intervention effects have not been explored thoroughly, only a few studies included a post-intervention follow-up in combination with measurements at the end of the intervention [30,34,38,42], making it difficult to draw conclusion about the long-term effects of interventions to promote physical activity among adolescents.

### Limitations and strengths

In order to collect the material for this review we developed a protocol in line with the instructions and the framework provided by the Cochrane Collaboration. We limited our search to publications available in electronic bibliographic databases and by reference checking. We did not search conference proceedings, grey literature or any unpublished interventions but, nevertheless we believe that we have found the core work in this area.

Although randomized controlled trials are considered to provide the strongest evidence, these trials are not feasible in some contexts and the results are sometimes not broadly implementable [48-50]. Therefore, we found it important to include also less rigorous designs that allow us to frame young people's behaviour and responds to different interventions in their own personal context. Although less rigorous designs could be downgraded in some methodological areas they could provide important information for developing strategies that could be replicated with a stronger design.

The use of the ANGELO framework made it possible to show in a clear and convenient manner (1) the range of approaches that have been investigated to promote physical activity among European teenagers and (2) the gaps in our knowledge about the effectiveness of various approaches to promote physical activity that should be filled by future research.

Several studies did not describe the content and implementation of the intervention in detail. Furthermore, space restrictions imposed by the journals may result in the omission of most study details. As this review is only based on manuscripts themselves, it was hard to draw a picture of how the components of these interventions were conducted and implemented. In addition, we might not have identified all the intervention components, as the authors have not reported everything that was actually done. Given this fact, design papers are very helpful. It is not uncommon that the design and the results of an intervention are described in separate papers. Checking the reference lists of the retrieved articles, literature reviews and the publication lists of the authors and co-authors of the retrieved articles should have minimized omission of design papers.

Earlier reviews [18,51] underlined the need to develop socio-cultural and socio-economical appropriate interventions for diverse populations around the globe, rather than to conduct interventions merely depending upon evidence that is hard to generalise. This review gives an overview of the evidence of effective interventions in Europe. This focus makes it possible to apply the results to the different countries of Europe.

Most of the reviewed studies were conducted in diverse SES groups, but analyses stratified by SES were not done. The studies [24,35] that included teenagers from a social disadvantaged group and the study [27] that included only vocational and technical schools did not compare the effectiveness of their program with the results of the same intervention in a population with an average socioeconomical or educational level. Since the prevalence of physical inactivity is higher in lower socioeconomic groups and it is an important explanatory factor for socio-economic inequalities in health and even in mortality [52,53], empirical evidence is needed towards physical activity promotion strategies for preventing the socio-economic inequalities in unhealthy behaviour in adolescence.

In addition, research into primary care and community based interventions is required before we can address the generalisability of these types of programs.

## Conclusion

Based on the evidence identified by this review we have provided a detailed insight in the literature concerning the effectiveness of interventions to promote physical activity among European teenagers.

In summary, our six main findings:

(1) School-based interventions generally lead to short term improvements in physical activity levels.

(2) Improvements in physical activity levels by school-based interventions were limited to school related physical activity and there was no conclusive transfer to leisure time physical activity.

(3) Including parents appeared to enhance school-based interventions.

(4) The physical activity level of secondary school children increased under the support of peers and the influence of direct environmental changes.

(5) Inconclusive evidence was found for the assumption that a multi-component approach should produce synergistic results.

(6) When interventions aimed to affect more than one health behaviour the intervention appeared to be less effective in favour of physical activity.

Nevertheless, the results of this review also underscore the need for future work in this area. Our findings highlight major gaps in our knowledge about physical activity promotion programs for teenagers. More high quality research is needed considering (i) the effectiveness of parental involvement, (ii) partnerships with the wider community as a component of school-based interventions, (iii) whole community based interventions and (iv) interventions conducted in the primary care setting. In addition, studies should be conducted to examine the independent effects of changes in the physical, socio-cultural or political environment on the physical activity levels of teenagers. Furthermore, more attention should be given to the development and use of standardized and validated instruments to measure physical activity in future research. Finally, also sustainability of short term intervention effects should be a central topic in future research.

This review confirms that there is little evidence about strategies for preventing the development of socio-economic inequalities in physical activity in adolescence. Further research will be conducted by the European project TEENAGE that will incorporate some of the included studies in a re-analysis to determine which interventions best contribute to the prevention of socio-economic inequalities in physical activity and in three other health-related behaviours in teenagers.

## Competing interests

The authors declare that they have no competing interests.

## Authors' contributions

FDM conducted the literature search, carried out the article screening, synthesised the findings and drafted the initial manuscript. IDB participated in the review design, provided feedback during the process and contributed in writing the manuscript. FVL, HS and NL provided critical comments on the manuscript and helped to draft manuscript revisions. All authors read and approved the final version of the manuscript.

## Supplementary Material

Additional file 1 **Intervention settings and components according to the Angelo framework**. This file contains a summary table with the intervention settings and components of the included studies according to the Angelo framework. Click here for file

Additional file 2 **Intervention characteristics, main results and results of the quality assessment of the included studies**. This file contains a summary table with a brief description of the methodological details of the interventions, such as study design, target population, relevant outcomes related to behaviour change, duration of the intervention, the main results and the ratings of the quality assessment process. Click here for file
