# Supplementary material for: Interventions for promoting physical activity among European teenagers: a systematic review
Source: Int J Behav Nutr Phys Act. 2009 Dec 6;6:82. doi: 10.1186/1479-5868-6-82 (PMC2795736; doi:10.1186/1479-5868-6-82)
Supplement: Additional file 1 — Intervention settings and components according to the Angelo framework. This file contains a summary table with the intervention settings and components of the included studies according to the Angelo framework. [file 1479-5868-6-82-S1.PDF]

Table 1 │ Intervention settings and components according to the Angelo framework

School setting					
	Individual component	Environmental components			
		Physical environment	Socio-cultural environment	Economical environment	Political environment
**Haerens et al. (2007) [23]**<br>C: no intervention	- Computer tailored intervention during classes with personal PA advice to encourage a physically active lifestyle.				
**Verstraete et al. (2006) [31]**<br>C: no intervention	- Each class group received 'activity cards' including examples of games and activities that can be performed with the game equipment. The equipment and the cards were presented by a research staff member.	- During lunch break and morning recess each class group received a set of game equipment.<br>*- The teachers were advised to divide the game equipment into different sets and to exchange those sets regularly.*	- The children were stimulated by the teachers to play with the equipment.	- Each class group received a free set of equipment.	
**Hill et al. (2007) [28]**<br>C: no intervention but they received an exercise word search	*3 experimental conditions:*<br>1. Intervention leaflet: the students received an exercise-promotion leaflet designed to raise awareness of different types of exercise, to promote positive attitudes, to target normative beliefs, to enhance behavioural control and to prompt exercise intentions.<br>2. Intervention leaflet + motivational quiz.<br>3. Intervention leaflet + implementation intention prompt: the students were encouraged to record a new additional goal for the coming week, stating what, when and how.				
**Tsorbatzoudis et al. (2005) [30]**<br>C: usual curriculum	- Teacher led health education program in PE context to support cognitive, emotional and behavioural components of the subjects' attitude towards physical activity. Three 45 min lectures were included to provide basic knowledge and tips followed by an interactive discussion and a question-and-answer session. The students received the presentation material and written messages. Weekly posters were put on the announcement board and leaflets concerning opportunities to participate in out of school PA were provided during stretching.<br>*- The content of the leaflet and the values recommendations were explained in two 2-hr seminars to the teachers.*<br>*- The teachers were instructed to accentuate values and standards connected with health improvement and exercise behaviour during their classes.*				
**Lubans and Sylva (2006) [38]**<br>C: own activity program as a part of the usual curriculum (2 sessions/ week)	- Researcher led health and exercise program to encourage change in PA by increasing individual knowledge, the confidence to be active and by developing techniques to enhance social support. Behavioural factors were enhanced through goal-setting techniques and the use of training diaries. The exercise program: 1x/week a researcher led workout (didactic component and participation in practical lifetime exercise activities) and 1x/week an own training session.<br>- Both groups received exercise information cards with details of sample training session.				
**Murphy et al. (2006) [40]**<br>C: were asked to maintain their normal PA behaviour	*2 experimental conditions:*<br>1. Teacher led PA program + the students received information about skills to manage their own PA behaviour.<br>2. Traditional self-led osteogenic PA program.<br>- Both groups received an education session about nutrition, exercise and bone health and discussed exercise adherence strategies and goal-setting. Great care was taken to foster self-efficacy: they were taught how to perform all exercises safely and optimally and how to monitor exercise intensity. The students recorded their daily PA in an activity diary.		- The diaries were discussed with the teachers to provide reinforcement on the activity diary.		
**Lindberg et al.**	- Teacher led health education program 'An adventure with				

**(2006) [37]**<br>C: usual curriculum	Pelle Pump' to increase knowledge on health and health behaviour, to support positive attitudes towards health and to support the development of health behaviour. The children received a study kit with theoretical and practical material on health behaviour and heart-lung function.<br>*- The teachers were free to use the study material so timetabling within schools varied. They could follow education seminars and they received a manual and booklets including theoretical and practical material.*				
**Chatzisarantis and Hagger (2005) [33]**<br>C: own intervention programme	- Intervention group: the students were asked to study a message targeting modal salient behavioural beliefs.<br>- Control group: the students were asked to study a message targeting nonsalient behavioural beliefs.<br>- Before the intervention, both groups were asked to read a definition of leisure-time PA, to give examples and to ask questions about the distinction between leisure-time and school-time PA. Both groups were asked to participate in PA during leisure time: 4 days/week at least 40 min., during the next 5 weeks.				
**Digelidis et al. (2003) [34]**<br>C: no intervention	- Teacher-led health education program with theoretical lessons that connect health and exercise to increase the knowledge and to emphasize positive feelings during exercise and the importance of positive social relations.<br>- The students were taught specific protocols to avoid time wasting during transition from one station to another during PE. The students had choices in each station to maximize self-determination.<br>- Personal goal setting programme.<br>*- The teachers had the freedom and the autonomy to choose the unit or the lesson that they wanted to teach. They were guided and supported by the researchers: they received designs of lesson plans, there were two 6-hr seminars, meetings and regular communication.*		- The teachers were instructed to emphasize with their students through discussion, values connected with task orientation and to provide evaluative feedback.<br>- The students were organized in groups of 3 where one of them had to give feedback.		- Change in PE: exercise in stations with task cards for each station, more goal-oriented activities, reciprocal style of teaching, low % of competitive tasks, self-talk, mental imagery and relaxation techniques into several activities.<br>- The teachers were instructed to use criteria of individual progress and mastery to provide evaluative feedback.

School setting (S) with involvement of family (F)

	**Individual component**	**Environmental components**			
		**Physical environment**	**Socio-cultural environment**	**Economical environment**	**Political environment**
**Harrison et al. (2006) [24]**<br>C: usual curriculum	- Teacher-led health education program: content lesson 2-5: self-reflection, identification of alternatives for TV-viewing; lesson 4: self-monitoring, goal setting, budgeting of time, selective viewing and the use of an 'activity point system' in conjunction with a diary to record activity/screen time; lesson 6-9: increasing PA and an attempt to revive traditional playground and street games. The students received workbooks. (S)<br>*- The teachers received learning objectives, training, a 3h briefing and educational materials for each lesson. Once every 2 weeks the schools were visited to offer support and check compliance. (S)*		- The parents were encouraged to support their children and to verify behaviour by signing the diaries. (F)		
**Haerens et al. (2007-2006) [27,43]**<br>C: no intervention	*Intervention without parental support:*<br>- Once each school year the students received a computer-tailored intervention for PA with personal activity advice. (S)<br>- The students received folders with information about their fitness level and possible ways to improve it after a physical fitness test. (S)	*Intervention without parental support:*<br>- Extra sport materials were made available. (S)<br>- More opportunities to be physical active at noon, during break, on Wednesday afternoons or after school hours. (S)<br>- More variation in the content of the activities (e.g. non-competitive activities were included to reach less skilled students). (S)<br>- The schools were encouraged to organize extra supportive activities beyond those planned in the intervention manual. (S)<br>*Additionally for the intervention with parental support:*<br>- The parents received information published in school papers and newsletters, an informative folder and a free CD with the adult computer-tailored intervention. (F) They were invited for an interactive	*Intervention without parental support:*<br>- The Schools were stimulated to encourage active transportation. (S)<br><br>*Additionally for the intervention with parental support:*<br>- The Parents were informed that their child completed the same programme and they		*Intervention without parental support:*<br>- More extra-curricular PA and variation in the activities. (S)

		meeting on healthy food, physical activity and the relationship with health and weight. (F)<br>*- The schools were guided and supported by research staff to get started. The 2nd intervention year they had to continue more independently. (S)*<br>*- In each school a work group was set up: they received information, guidelines, an intervention manual and educational material. (S)*	were asked to discuss their results and give their child support to create a healthier lifestyle. (F)		
**Christodoulos et al. (2006) [26]**<br>C: usual curriculum	- Teacher delivered health education program combining weekly classroom lectures to increase pupils' knowledge, health education integrated into other school subjects, computer aided lessons to promote pupils' interaction and entertainment, information about sports activities and a 3 min talk about PA and health in each PE lesson. (S)<br>- Individualized goal setting programme. (S)<br>- The teachers received teacher-oriented seminars and teaching material. (S)		- Information was disseminated about community based sports programmes to promote extra-curricular PA. (S)<br>- Parental involvement and encouragement through homework assignments with family activities. (F)<br>- The parents received educational material and PA and nutritional guidelines. (F)		- Change in PE: more cooperative, enjoyable, fitness oriented and goal oriented activities were used. The design of the lessons allowed every student to choose a level of difficulty. (S)<br>- Integration of health education into several school subjects. (S)
**<u>School setting (S) with involvement of family (F) and community (C)</u>**					
	**Individual component**	**Environmental components**			
		**Physical environment**	**Socio-cultural environment**	**Economical environment**	**Political environment**
**Simon et al. (2004-2006) [29,44]**<br>C: no intervention	- Theoretical lessons and debates to increase skills, knowledge, attitudes, beliefs and motivation towards PA and an active lifestyle. (S)	- Change in physical, structural, institutional conditions for PA that encourage to use the knowledge and skills they have learned.(S)<br>- Sporting events and active transport were organised and a mean of 10 different weekly activities was provided.(S)<br>- New opportunities were organized during and after school hours taking into account the barriers to being active. (S).<br>- Transfer to the PA areas have been organized and supplementary busses were made available when necessary.(C)<br>*- The intervention staff was informed and trained to work with the different partners.(S)*<br>*- The ICAPS coordinators regularly visited intervention school members to help resolve material or personnel needs.(S)*	- Social support by PA instructors, teachers, peers.(S)<br>- Support by parents to enhance the PA level but also by letting them walk or cycle to school. (F)<br>- Policy makers of local communities were requested to provide a supportive environment that promotes enjoyable PA. (C)<br>- Media diffusion to reinforce participants engagement. (C)	- Free of charge entry to safe accessible facilities. (C)	- More extra-curricular PA with new opportunities, variation and change in content; emphasis on fun and pleasure, well-being, the company of others and an absence of competitive aspects. (S)<br>- All teachers were invited to propose various ways of integrating PA and to have PA during their classes.(S)<br>- Partnerships with school boards, teachers, medical staff (S), territorial and community agencies in charge of recreational areas, transportation infrastructure, club educators.(C)<br>- Change in public transport: transfer to PA areas and supplementary busses. (C)<br>- Reflections on the development of bikeways around the schools. (C)
**Jurg et al. (2006) [35]**<br>C: no intervention	- Card game with assignments in the class and at home to raise awareness on the importance of PA for health and one's own PA behaviour, self-efficacy, social support and planning skills of the children and the parents. (S)	- More and easy accessible school PA opportunities in or near the school (e.g. school sport clubs, once a year an activity week). During school hours the children get acquainted with a variety of sports, each sport a number of times in several weeks. (S)<br>- Regular breaks for PA, relaxation and posture exercises: 'The class moves' consists of calendars containing exercises separated on 10 themes, each for every school month. (S)<br>*- At least once a year a parental information service was organized in which the importance of PA and sports for children and the role of parents to support and stimulate was emphasized. (F)*<br>*- The teachers received training to use 'the class moves.' (S)*	- The PE teacher monitored the pupils once a year in order to stimulate pupils in a structured way in their development in sport and PA and in attaining the PA recommendation for youth. (S)<br>- Support of peers and friends in the classroom. (S)<br>- Support of family members. (F)		- 'School sport activities' were designed to be adopted in the regular school policy in order that they would be available all school year long. (S)<br>- Change of PE. (S)<br>- PA breaks. (S)<br>- Partnerships with municipal authorities, local sport services and local sport clubs. (C)
**Moon et al. (1999) [39]**<br>C: no intervention	- Comprehensive, coordinated, cross-curricular health education programme to influence the health-related knowledge, attitude and behaviour of the pupils.(S)	- Change in a healthy physical environment.(S)<br>*- The staff received health related information and in-service training.(S)*<br>*- The Healthy school Award scheme provided structured frameworks, health-related targets and external support.(S)*	- Health promoting initiatives. (S)<br>- Change of ethos. (S)<br>- Involvement of parents/family in children's health education. (F)<br>- Community health promotion supported by Health Authority or Local education Authority. (C)	- Financial support was provided and varied from school to school. (C)	- Change in health promotion policy and practice with 9 key areas: the curriculum, links with the community, smoke-free-school, healthy food choices, PA, responsibility for health, health promoting workplace, environment, equal opportunities and access. (S)<br>- New teaching methods with actively involved students in their learning and the decision making process and focus on

	Individual component	Environmental components: Physical environment	Socio-cultural environment	Economical environment	Political environment
					them as individuals and their needs.(S)<br>- Change in curriculum: comprehensive, coordinated, cross-curricular programme throughout the school career<br>- Each school identified a coordinator.(S)
**Community (C) with involvement of schools (S)**					
	**Individual component**	**Environmental components**			
		**Physical environment**	**Socio-cultural environment**	**Economical environment**	**Political environment**
**Baxter et al. (1997) [25]**<br>C: no intervention	- Curricular activities on general heart health and exercise. (S)<br>- Health days with information on lifestyle issues. (S)<br>- Action Heart worker facilitated activities targeting individual risk behaviours.(S)<br>- Peer-led health education: health days runned by a number of young people for which they undertook a training programme. (S)	- Change of school environment.(S)<br>- Activities targeting individual risk behaviours. (C)<br>*- The staff and the schools received training and were supported. (S)*<br>*- The interventions used were informed largely by the expert knowledge base of the health service staff involved.(S)*	- General health promotion activities in the schools and the community. (S)+(C),<br>- Publicity with leaflets, posters, T-shirts and other promotional material. (S)+(C)<br>- Peer led health education. (S)	- The schools were provided with resources. (S)<br>- Printing costs were met by the action heart budget.(S)	- New / change in policies.(S)+(C)<br>- Action Heart Charter: health promotion contract between Action Heart, the schools and organisations in the intervention area. It itemises certain health action points which they agreed to undertake with the support of the action heart team. (S)+(C)
**Primary care**					
	**Individual component**	**Environmental components**			
		**Physical environment**	**Socio-cultural environment**	**Economical environment**	**Political environment**
**Ortega-Sanchez et al. (2004) [41]**<br>C: no intervention	- Depending on their current activity level the adolescents received reinforcement counselling to maintain their current level of activity, increase counselling to increase their current level of activity or initiation counselling to begin exercise based on ask-assess-advice principle.<br>*- The physicians received written guidelines on how to provide the three types of counselling.*				
**Walker et al. (2002) [42]**<br>C: no intervention	- General practice consultation: consultation with the practice nurse to discuss health and health related behaviour focusing on topics of their choice. Participants who did not attend received health promotion leaflets at home.<br>*- The nurses received training in the study protocol.*				
**Kelleher et al. (1999) [36]**<br>no C	- A 10 min. counselling session with background educational material in an so-called opportunistic or recall clinic run by a doctor or a nurse. The subjects received an information card concerning diet, smoking and exercise based on current national guidelines. Opportunistic clinics: the subjects received a lifestyle session when they visited the surgery for other reasons or were invited back for a special session. The recall practices: received an invitation by letter.<br>*- The nurses and practitioners received training.*				
**Individual**					
	**Individual component**	**Environmental components**			
		**Physical environment**	**Socio-cultural environment**	**Economical environment**	**Political environment**
**Woods et al. (2002) [32]**<br>C: no intervention	- Personally mail-delivered self-instructional messages, made up of 2 packages on active living. PAL1: targeting consciousness raising and social liberation. PAL2: targeting self re-evaluation, self-liberation, counter-conditioning, helping relationships and reward management.				

(S) = school; (F) = family; (C) = community
PA = physical activity
PE = physical education
