# Supplementary material for: Interventions for promoting physical activity among European teenagers: a systematic review
Source: Int J Behav Nutr Phys Act. 2009 Dec 6;6:82. doi: 10.1186/1479-5868-6-82 (PMC2795736; doi:10.1186/1479-5868-6-82)
Supplement: Additional file 2 — Intervention characteristics, main results and results of the quality assessment of the included studies. This file contains a summary table with a brief description of the methodological details of the interventions, such as study design, target population, relevant outcomes related to behaviour change, duration of the intervention, the main results and the ratings of the quality assessment process. [file 1479-5868-6-82-S2.PDF]

Table 2│ Intervention characteristics, main results and results of the quality assessment of the included studies

**School setting**												
**Intervention details**						**Quality assessment**						
**Study/ country**	**Study design**	**Sample characteristics**	**Duration/ measurements**	**Outcomes**	**Main results**	**Selection bias**	**Study design**	**Control for confounders**	**Blinding**	**Data collection**	**Withdrawals and dropouts**	**Global rating**
**Haerens et al. (2007) [23] Belgium**	CRCT	n = 281<br>10 schools<br>mean age 13,2 (SD 0,7)<br>48% boys	1 hour<br>- baseline<br>- 3 months after intervention	- total PA level (TPA) (min/day): school related PA index (school PA) (active transportation + extracurricular activities) + leisure time sport index (LTS) and leisure time active transport index (LTAT)	total PA: ↔<br>school PA: +<br>LTS: ↔<br>LTAT: ↔	***	***	***	**	***	***	***
**Verstraete et al. (2006) [31] Belgium**	CRCT	n = 249<br>7 schools<br>I: mean age 10.8 (SD 0.6) (final sample)<br>C: mean age 10.9 (SD 0.7) (final sample)<br>49% boys	3 months<br>- baseline<br>- 3 months after baseline	- total activity counts: light (LPA), moderate (MPA) and vigorous intensity activity (VPA) during morning recess and lunch break (% of recess time and lunch break)<br>- MVPA engagement: moderate + vigorous intensity activity (% of recess time and lunch break)	morning recess: LPA: + (♀)<br>MPA:+++ (♀)<br>VPA: ↔<br>MVPA:++ (♀)<br>lunch break LPA: +++<br>MPA: +++<br>VPA: +++<br>MVPA: +++	**	***	*	**	***	***	**
**Hill et al. (2007) [28] UK**	CRCT	n = 620<br>22 classes<br>1 school<br>mean age (final sample) 16.97 (SD 1,4)<br>49% boys	20 min.<br>- baseline<br>- 3 weeks after intervention	- average number of exercise sessions for at least 30 min/week during or after school without PE	exercise sessions: ++ in L, LQ, LII	**	***	**	**	*	***	**
**Tsorbatzoudis (2005) [30] Greece**	CT	n = 366<br>4 schools<br>mean age 14,2 (SD 0,69)<br>48% boys	12 weeks (36 PE lessons)<br>- baseline<br>A. end of the intervention<br>B. 4-6 weeks after intervention (subsample)	- exercise habits (score)	A. exercise habits: +++<br>B. exercise habits: ↔	**	***	**	**	***	*	**
**Lubans and Sylva (2006) [38] UK** 'Lifetime Activity Program'	RCT	n = 78 individuals who elected to participate in an unit on health and fitness rather than the traditional team sports offered at school<br>mean age 16,7 (SD 0,5); 38,5% boys	10 weeks (2x/week)<br>- baseline<br>A. end of intervention<br>B. 3 months after intervention	- moderate to vigorous PA (min/week) of 20 min. or longer (MVPA)	A.<br>MVPA: +++<br>B.<br>MVPA: ↔	*	***	***	*	***	***	*
**Murphy et al. (2006) [40] Ireland**	CRCT	n = 90 sedentary girls<br>5 schools<br>mean age 16,3 (SD 0,6)	6 months<br>- baseline<br>A. end of intervention<br>B. 1 month after intervention (subsample, process evaluation)	- daily PA: duration (hours/week) and intensity of PA. (above 13 on the BORG RPE scale: moderate to vigorous PA)	A. in both I groups:<br>(P-value NR)<br>duration: (+)<br>intensity: (+)	**	***	*	**	*	***	*
**Lindberg et al. (2006) [37] Sweden** 'An adventure with Pelle Pump'	Crosse ctional	n = 1369<br>mean age<br>I:12,5 (SD 0,60)<br>C: 12,5 (SD 0,50)<br>50% boys	3 months<br>- after 2 year program	- PA index (score)	PA index: ↔	**	*	*	***	**	***	*
**Chatzisarantis and Hagger (2005) [33] UK**	RCT	n = 83<br>2 schools<br>mean age 14,60 (SD 0,47)<br>49,5% boys	5 min<br>- baseline<br>- after 5 weeks	- PA during leisure time: frequency of mild, moderate and vigorous PA in the last 5 weeks	PA participation: ↔	**	***	**	*	***	*	*
**Digelidis et al. (2003) [34] Greece**	CT	n = 783<br>4 I, 19 C schools<br>I: mean age 11,88 (SD 0,60); 50% boys<br>C: mean age 12,14 (SD 0,77); 47% boys	one school year (3x/week, 45 min)<br>- baseline<br>A. end of intervention<br>B. 10 months after intervention	- behaviour: frequency of regular exercise in the previous month: regular exercise: intensive out of school exercise at least 2x/week for 1 hour or more	A.<br>PA behaviour: ↔<br>B.<br>PA behaviour: ↔	*	***	**	*	*	*	*

School setting with involvement of family

Study/ country	Study design	Sample characteristics	Duration/ measurements	Outcomes	Main results	Selection bias	Study design	Control for confounders	Blinding	Data collection	Withdrawals and dropouts	Global rating
**Intervention details**						**Quality assessment**						
**Harrison et al. (2006) [24] Ireland**<br>'Switch off – get active'	CT	n = 312<br>9 schools in areas of social disadvantage<br>mean age 10,2 (SD 0,70)<br>57% boys	16 weeks<br>(10 lessons/30 min)<br>- baseline<br>- end of intervention	- moderate to vigorous PA (MVPA): principal PA + intensity (30 min blocks/day)	MVPA: +	**	***	***	**	***	***	***
**Haerens et al. (2006, 2007) [27,43] Belgium**	CRCT	n = 2840<br>15 schools<br>mean age 13,1 (SD 0,8)<br>63% boys<br>32.5% of higher SES	2 school years<br>- baseline<br>A. end of first school year<br>B. end of intervention	- total PA level (TPA) (min/day): school-related PA index (school PA) + leisure time PA index (LTPA) (leisure time sport (LTS) + leisure time active transportation (LTAT))<br><br>*Subsample*<br>- accelerometer data: sedentary, PA of light intensity (LPA) and PA of moderate to vigorous intensity (MVPA) (min/day)	A. School PA: + (I and I+P)<br>LTAT: +++ (♀) (I)<br>TPA: ↔<br>LTS: ↔<br>Accelerometer data:<br>LPA: + (I and I+P)<br>MVPA: + (I+P)<br>meeting guidelines: ↔<br>B. School PA: + (♂)<br>LTPA: ↔<br>Accelerometer data<br>sedentary: ↔<br>LPA: +++ (♂); + (♀)<br>*MVPA: (+ ♂trend)*	**	***	***	**	***	**	**
**Christodoulos et al. (2006) [26] Greece**	CRCT	n = 78<br>2 schools<br>I: mean age 11,2 (SD 0,40)<br>C: mean age11,2 (SD 0,30)<br>54% boys	1 school year (two 45 min PE classes /week)<br>- baseline<br>- end of intervention	- duration, frequency and total time spent on total moderate to vigorous PA and organised moderate to vigorous PA: weekly leisure supervised activities out of school (hours/week TMVPA, OMVPA)<br>- % meeting the guidelines:60 min/day of moderate to vigorous PA	OMVPA: +<br>TMVPA: ↔ *(trend)*<br>meeting the guidelines: +	**	***	***	**	***	*	**

School setting with involvement of family and community

Study/ country	Study design	Sample characteristics	Duration/ measurements	Outcomes	Main results	Selection bias	Study design	Control for confounders	Blinding	Data collection	Withdrawals and dropouts	Global rating
**Intervention details**						**Quality assessment**						
**Simon et al. (2004-2006) [29,44] France**<br>'ICAPS'	CRCT	n = 954<br>8 schools<br>mean age 11,7(SD 0,6)<br>46,3% I ; 51,8% C boys.	4 school years<br>- baseline<br>- 6 months of intervention	- leisure organised PA in and outside sport clubs (%)	LOPA: ++	***	***	***	*	***	***	**
**Jurg et al. (2006) [35] The Netherlands**<br>'JUMP-in'	CT	n = 510<br>6 schools (4 I, 2C)<br>49% I, 48% C boys<br>9-12 years<br>I: 71% ,C: 94% foreign etnicity;	1 school year<br>- baseline<br>- end of intervention	- total PA score: min/day at least moderately active: daily activity score: PA score in moderate active min/day (MET-score ≥ 5) + sports score: total amount of min/day organized sports.<br>- meeting the guidelines: 60 min/day of moderate PA	total PA score: +<br>grade 4-5: ↔<br>grade 6: +++<br>meeting the guidelines: +<br>grade 4-5: ↔<br>grade 6: +++	**	***	***	*	*	*	*
**Moon et al. (1999) [39] UK**<br>'Wessex Healthy School award Scheme'	two group pre-post design	n = 16 schools; pupil numbers ranged from 440 to 1486<br>mean age NR<br>11-16 years<br>% boys NR	4 school terms (15 months)<br>- baseline<br>- end of intervention	- PA behaviour : % taking part in sports at school (not PE) once or more a week	PA behaviour: ↔	**	**	*	*	*	**	*

**Community with involvement of schools**												
			**Intervention details**				**Quality assessment**					
**Study/ country**	**Study design**	**Sample characteristics**	**Duration/ measurements**	**Outcomes**	**Main results**	**Selection bias**	**Study design**	**Control for confounders**	**Blinding**	**Data collection**	**Withdrawals and dropouts**	**Global rating**
**Baxter et al. (1997) [25]**<br>**UK**<br>'Action Heart'	CT	1991: n = 2685<br>1994: n = 3224<br>4 schools<br>11 and 14 years (years 7 and 10)	3 years<br>- baseline<br>- end of intervention	- exercise: % of the students that exercise 3 or more times weekly	exercise: ↔<br>(I increased with 4%, C remained stable, p-value NR)	**	***	**	**	**	***	**
**Primary care**												
			**Intervention details**				**Quality assessment**					
**Study/ country**	**Study design**	**Sample characteristics**	**Duration/ measurements**	**Outcomes**	**Main results**	**Selection bias**	**Study design**	**Control for confounders**	**Blinding**	**Data collection**	**Withdrawals and dropouts**	**Global rating**
**Ortega-Sanchez et al. (2004) [41]**<br>**Spain**	RCT	n = 448<br>mean age 17,0 (SD 2,4)<br>58 % boys	3 x 5-10 min (baseline, 6 and 12 month visit)<br>- baseline<br>A. 6 month after 1st session<br>B. 12 month after 1st session	- proportion of actives: % of the sample<br>- duration (min/week), frequency (days/week) and intensity in points (mild = 1, moderate = 2, vigorous = 3)	A. proportion of A: ++<br>duration: +<br>frequency:++<br>intensity: ++<br>B. proportion of A: ++<br>duration: ++<br>frequency:++<br>intensity: +++	**	***	**	*	*	**	*
**Walker et al. (2002) [42]**<br>**UK**	RCT	n = 1488<br>eight general practitioners<br>mean age 14,8<br>49% boys	20 min<br>- baseline<br>- 3 months after intervention<br>- 12 months after intervention	- prevalence of health related behaviour (PA): % of teenagers who reported positive behaviour change	A. PA behaviour: ↔<br>B. PA behaviour: ↔	**	***	*	*	*	*	*
**Kelleher et al. (1999) [36]**<br>**Ireland**	one group pre-post design	n = 203 (8-11 years);<br>n = 111 (12-15 years),<br>n = 196 (adults)<br>mean age NR<br>47 % boys	10 min.<br>- baseline<br>- 1 year after intervention	- lifestyle characteristics: exercise: times/week,	exercise behaviour: ↔	**	*	*	*	*	**	*
**Individual**												
			**Intervention details**				**Quality assessment**					
**Study/ country**	**Study design**	**Sample characteristics**	**Duration/ measurements**	**Outcomes**	**Main results**	**Selection bias**	**Study design**	**Control for confounders**	**Blinding**	**Data collection**	**Withdrawals and dropouts**	**Global rating**
**Woods et al. (2002) [32]**<br>**Scotland**	RCT	n = 459<br>mean age 19 (SD 4,5)<br>38% boys	PAL1 distributed in Jan., PAL 2 in Nov.<br>- baseline<br>- 7 months after baseline	- exercise behaviour: membership of the Sport and Recreation Service at the university (%)	exercise behaviour: +++	**	***	***	**	***	*	**

+++ = p≤0.001
++ = p≤0.01
+ = p≤0.05
↔ no significant effect (I vs C), sustained effect
*** = strong
** = moderate
* = weak

I: intervention group
C: control group
PA: physical activity
PE: physical education
NR: not reported
NA: not applicable
